# Supplementary material for: Clustering huge protein sequence sets in linear time
Source: Nat Commun. 2018 Jun 29;9:2542. doi: 10.1038/s41467-018-04964-5 (PMC6026198; doi:10.1038/s41467-018-04964-5)
Supplement: Supplementary file 1 — Supplementary Information [file 41467_2018_4964_MOESM1_ESM.pdf]

Clustering huge protein sequence sets in linear time

Martin Steinegger[1,2,3] **& Johannes Söding**[1,*]

[1]Quantitative and Computational Biology group, Max-Planck Institute for Biophysical Chemistry, Am Fassberg 11, 37077 Göttingen, Germany. [2]Department for Bioinformatics and Computational Biology, Technische Universität München, 85748 Garching, Germany, Department of Chemistry, [3]Seoul National University, Seoul, Korea

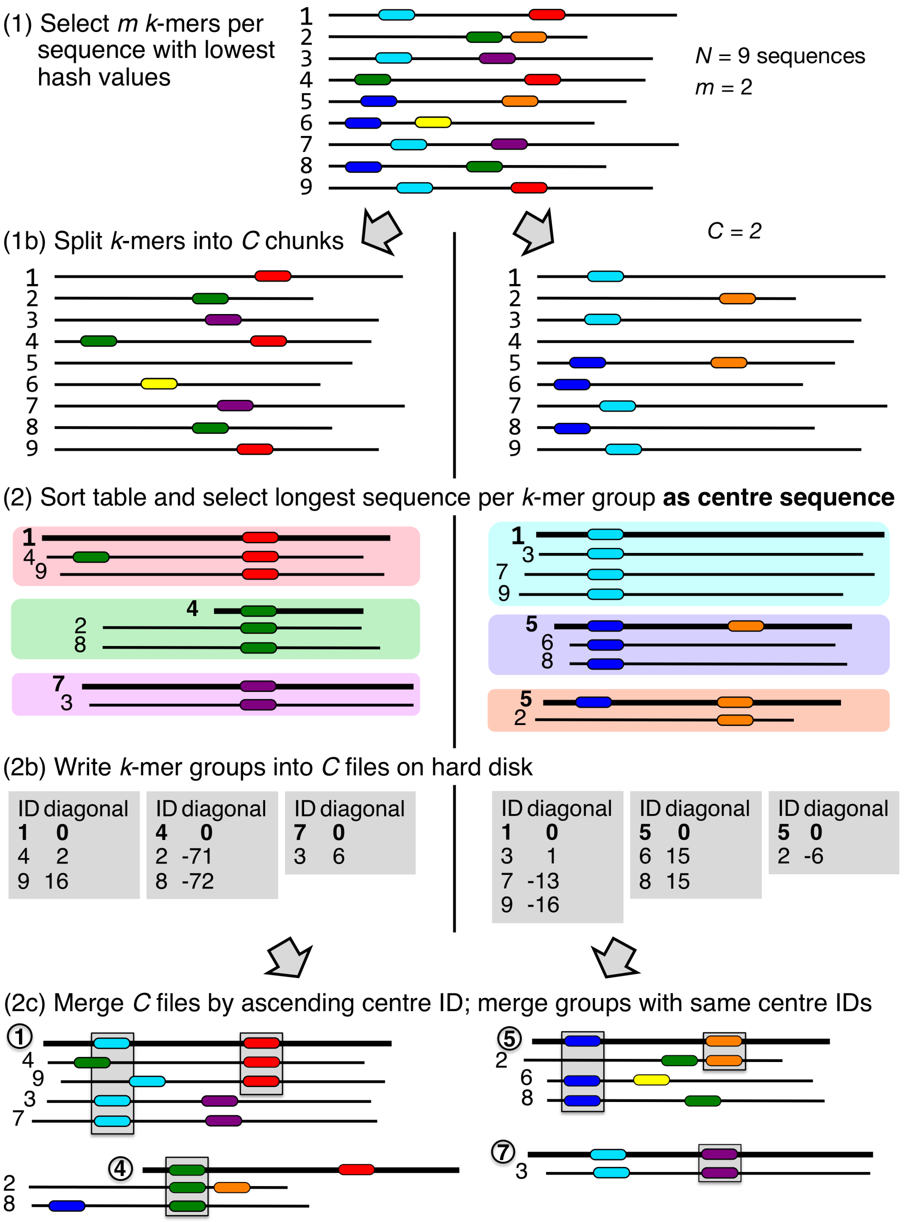

Supplementary Figure 1. **Splitting the database into chunks** For each chunk index $c \in \{0, \ldots, C-1\}$ we run Linclust steps 1 and 2 (Figure 1) normally but extract only k-mers whose numerical index modulo C yields a rest c. This way each of the C runs builds up a k-mer table with only about mN/C lines instead of mN, and hence each run needs C times less memory. Each run writes out a file with all found k-mer groups (sequence ID, diagonal). The diagonal is $i - j$ of the k-mer match, where i is the position of the groups k-mer in the centre sequence and j the position in the other sequence. Afterwards all C files are merged into a single file such that k-mer groups are sorted by ascending centre IDs. Finally, Linclust steps 3 to 5 are performed as usual.

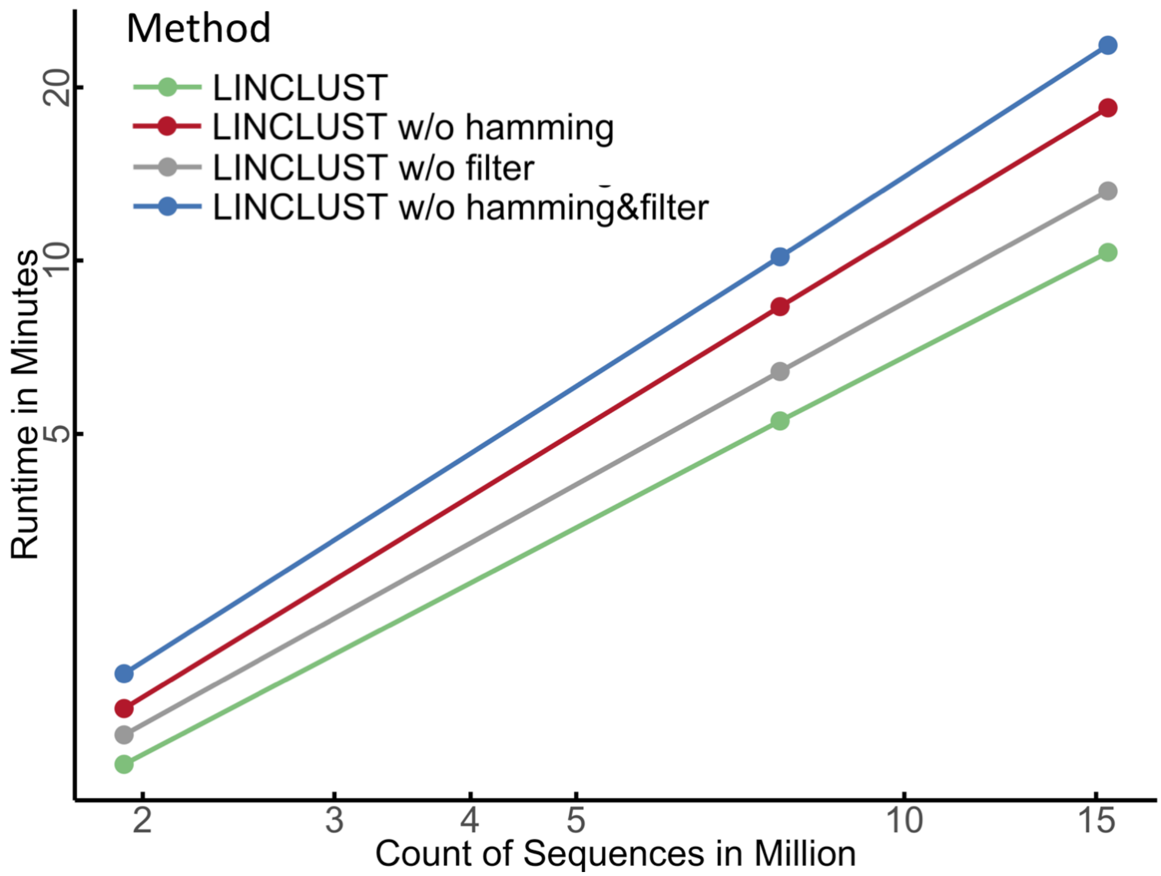

Supplementary Figure 2. **Contribution of Hamming distance pre-clustering and gapless local alignment filter steps to the decrease of the Linclust runtime** Double-logarithmic plot of runtimes versus sequence set size illustrating the contribution of runtime decrease for the Hamming distance pre-clustering and ungapped alignment filtering stage.

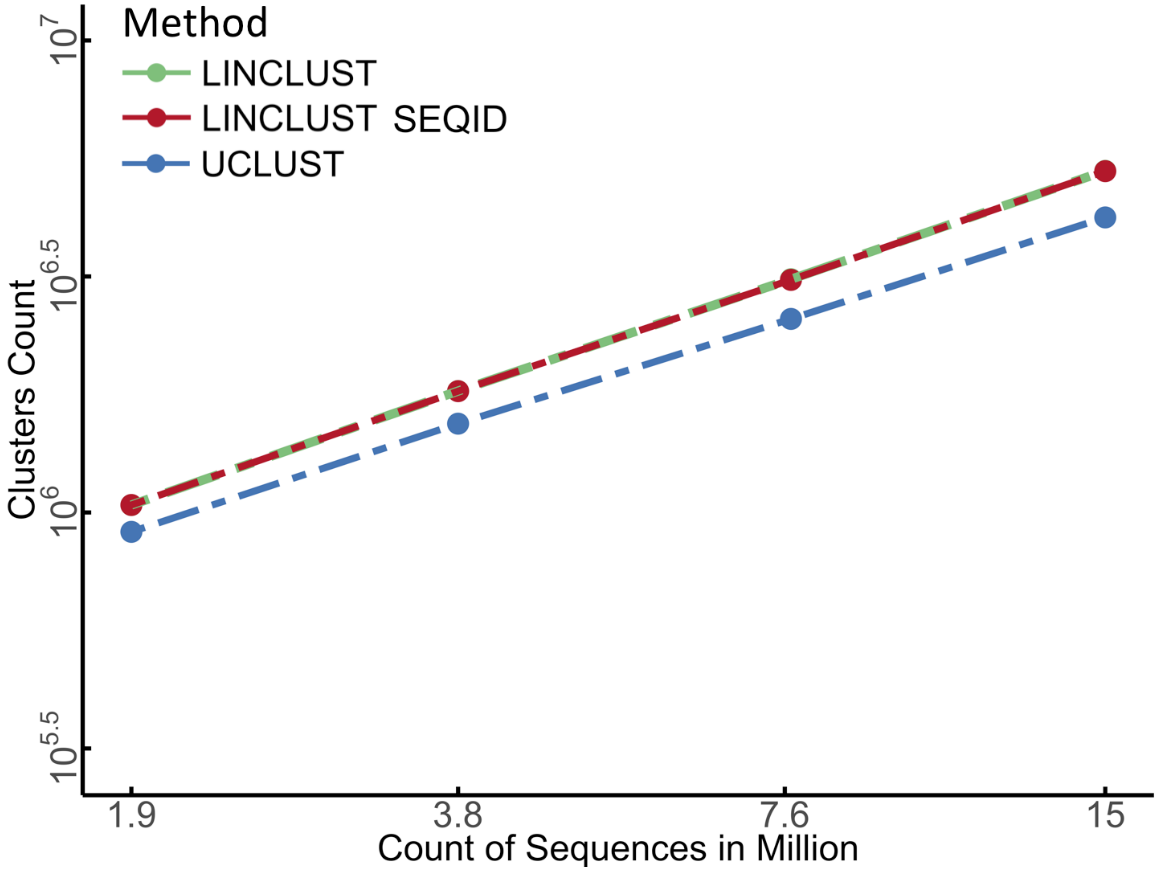

Supplementary Figure 3. **Equivalence of two sequence identity measures.** Number of clusters obtained at 50% sequence identity for Linclust with two different sequence identity definitions: (1) the default definition ("Linclust"), based on the local alignment score divided by the maximum length of the two aligned sequence segments; and (2) the fraction of identical residues in the alignment relative to the number of aligned columns including gaps ("Linclust seqid"). Both measures produce very similar numbers of clusters.

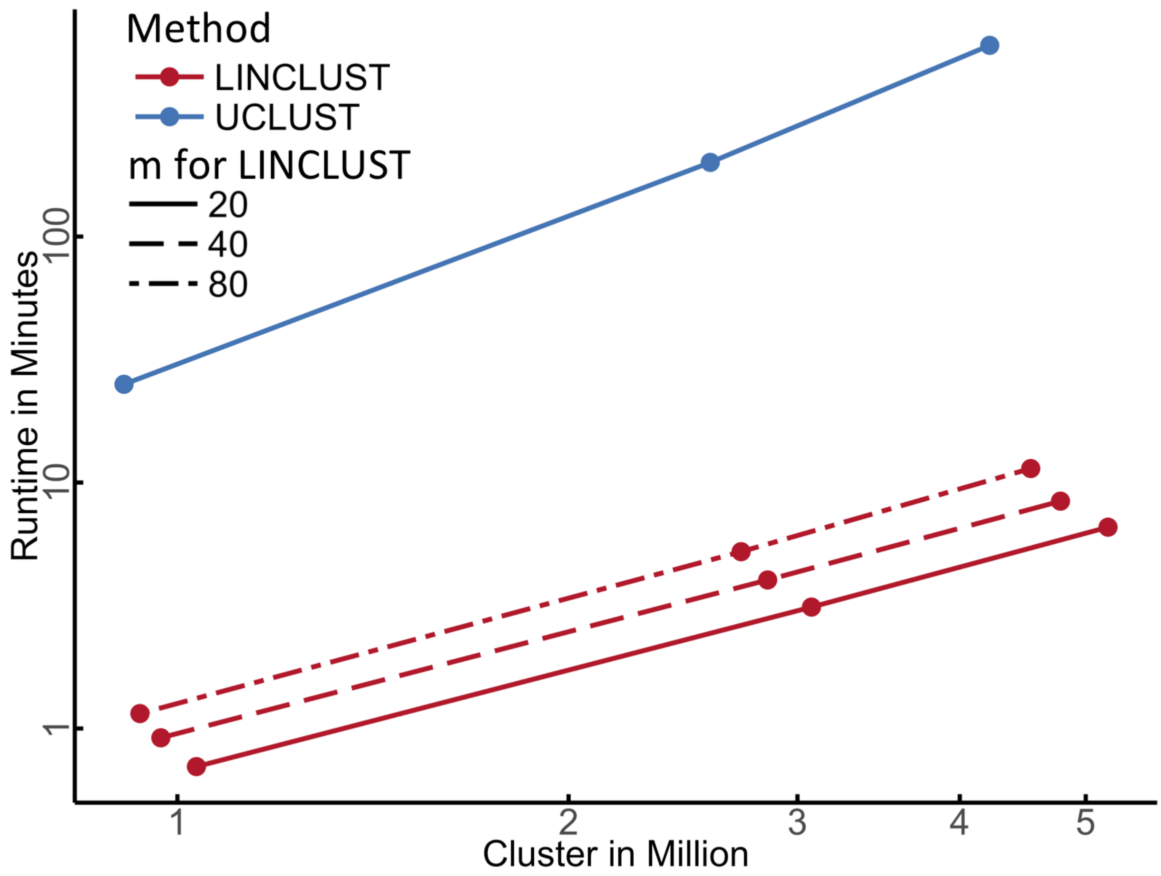

Supplementary Figure 4. **Influence of the number** m **of** k**-mers extracted per sequence.** Double-logarithmic plot of runtimes versus cluster size. Through the parameter m, the number of k-mers selected per sequence, the user can set the trade-off between sensitivity and speed. At $m = 80$ the runtime of Linclust increases by a factor of 1.66 over the default setting $m = 20$ while producing 8% fewer clusters. At $m = 80$, Linclust generates only 7% more clusters than UCLUST while still being much faster.

```c
// (c) 2017 Johannes Soeding & Martin Steinegger, Gnu Public License version 3
// Rotate left macro: left circular shift by numbits within 16 bits

#define RoL(val, numbits) (val << numbits) ^ (val >> (16 - numbits))

// Transform each letter x[i] to a fixed random number RAND[x[i]]
// to ensure instantaneous mixing into the 16 bits
// Do XOR with RAND[x[i]] and 5-bit rotate left for each i from 1 to k
unsigned circ_hash(const int * x, unsigned length){
 short unsigned RAND[21] = {0x4567, 0x23c6, 0x9869, 0x4873, 0xdc51, 0x5cff, 0x944a, 0x58ec,
                            0x1f29, 0x7ccd, 0x58ba, 0xd7ab, 0x41f2, 0x1efb, 0xa9e3, 0xe146,
                            0x007c, 0x62c2, 0x0854, 0x27f8, 0x231b}; // 16 bit random numbers
 short unsigned h = 0x0;
 h = h^ RAND[x[0]];                  // XOR h and ki
 for (int i = 1; i < length; ++i){
   h = RoL(h, 5);
   h ^= RAND[x[i]];                  // XOR h and ki
 }
 return h;
}

// Rolling hash variant for previous hash function:
// Computes hash value for next key x[0:length-1] from previous hash value
// hash( x[-1:length-2] ) and x_first = x[-1]
unsigned circ_hash_next(const int * x, unsigned length, int x_first, short unsigned h){
 short unsigned RAND[21] = {0x4567, 0x23c6, 0x9869, 0x4873, 0xdc51, 0x5cff, 0x944a, 0x58ec,
                            0x1f29, 0x7ccd, 0x58ba, 0xd7ab, 0x41f2, 0x1efb, 0xa9e3, 0xe146,
                            0x007c, 0x62c2, 0x0854, 0x27f8, 0x231b}; // 16 bit random numbers
 // undo INITIAL_VALUE and first letter x[0] of old key
 h ^= RoL(RAND[x_first], (5*(length-1)) % 16);
 // circularly permute all letters x[1:length-1] to 5 positions to left
 h =  RoL(h, 5);
 // add new, last letter of new key x[1:length]
 h ^= RAND[x[length-1]];
 return h;
}
```

Supplementary Figure 5. ***k*-mer hashing function implemented in C.**
